# Falsely Elevated CA 15-3 Levels in Ovarian Sarcoidosis with Peritoneal Involvement and Ascites

**DOI:** 10.1155/2018/2039730

**Published:** 2018-01-22

**Authors:** Sotirios Tsiodras, Alina-Roxani Gouloumi, Zoi Tsakiraki, George Chrelias, Charalambos Chrelias, Stylianos Loukides, Ioannis Panayiotides

**Affiliations:** ^1^4th Department of Internal Medicine, University of Athens Medical School, “Attikon” University Hospital, Athens, Greece; ^2^2nd Department of Pathology, University of Athens Medical School, “Attikon” University Hospital, Athens, Greece; ^3^3rd Department of Obstetrics and Gynecology, University of Athens Medical School, “Attikon” University Hospital, Athens, Greece; ^4^2nd Department of Pulmonary Medicine, University of Athens Medical School, “Attikon” University Hospital, Athens, Greece

## Abstract

A rare case of ovarian sarcoidosis with peritoneal and omental involvement presenting as an ovarian malignancy is presented. Clinical, laboratory, and radiological evaluation of the patient revealed ascites and high levels of serum CA 125 and CA 15.3. The diagnosis of sarcoidosis was confirmed with pathology findings on tissues obtained during surgical laparotomy. Establishing the diagnosis of sarcoidosis can be treacherous and was complicated in this case by the falsely elevated biomarkers and ascites.

## 1. Introduction

Atypical manifestations of sarcoidosis occurring outside typically affected organs make histology necessary in establishing the diagnosis after excluding infectious and neoplastic mimics of the disease. Extrapulmonary sarcoidosis has been reported in several sites; however localization of the disease in the female reproductive system is extremely rare accounting for less than 1% of cases [1]. Herein we present a rare case of ovarian sarcoidosis with peritoneal and omental involvement presenting as ovarian malignancy with falsely elevated cancer biomarkers.

## 2. Case Presentation

**Figure 1:**
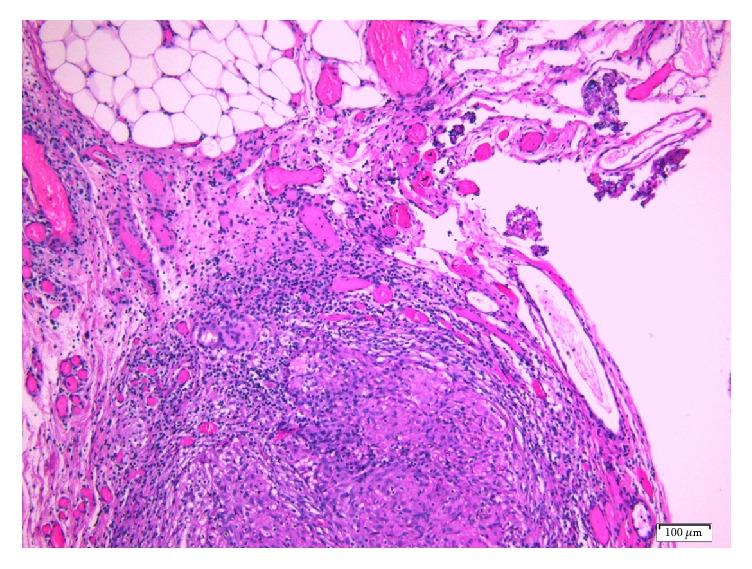
Omental adipose tissue containing a sarcoid-type granuloma (Hematoxylin and Eosin, ×10).

**Figure 2:**
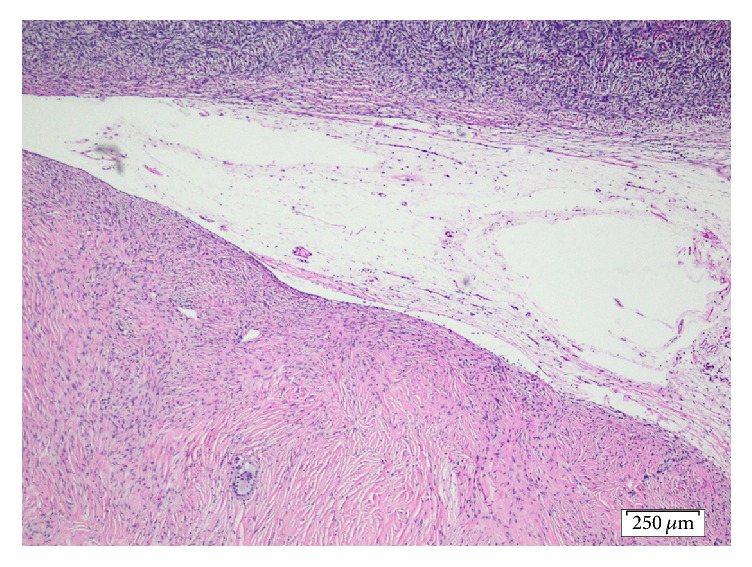
Left ovary (cortex on top) containing a fibrotic focus with a single multinucleated giant cell. (Hematoxylin and Eosin, ×4).

**Figure 3:**
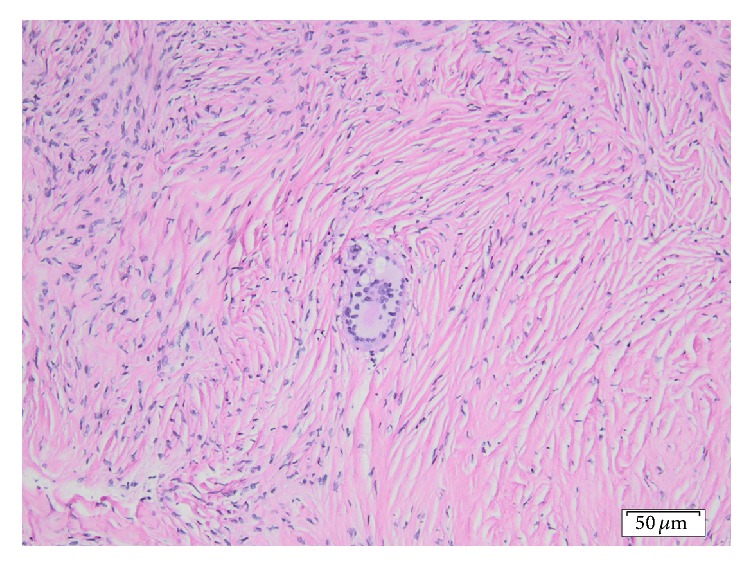
The single, Langhans-type multinucleated giant cell within the fibrotic focus of the left ovary (Hematoxylin and Eosin, ×20).

A 79-year-old, gravida 4, para 2, Caucasian female with a history of weight loss and fatigue for 4 months was referred to our hospital for investigation of her symptoms. Medical history was significant for dyslipidemia and hypertension treated with irbesartan. The physical examination disclosed no abnormalities. Due to concern for malignancy, diagnostic imaging was performed. Chest X-ray was negative. Ultrasound, CT, and MRI of the abdominal area showed the presence of minimal amounts of free liquid in the perihepatic area, in the paracolic sulci, in the right inguinal region, and in the pouch of Douglas, without the involvement of retroperitoneal, paraaortic, and iliac chain lymph nodes. Since the suspicion of malignancy remained high, the gynaecology team ordered cancer biomarkers; testing disclosed a CA 125 value of 225.20 U/ml (normal range < 35 U/ml) and a CA 15.3 value of 73.9 U/ml (normal values < 35 U/ml). Levels of carcinoembryonic antigen were within normal range; that is, CEA levels were 1.43 ng/ml (normal values < 3 ng/ml) and carbohydrate antigen 19-9 (CA 19-9) levels were 6,4 U/ml (normal values < 37 U/ml). Cytological examination of the ThinPrep® sample obtained via a transvaginal fine-needle aspiration was negative for malignancy only disclosing an abundance of histiocytes, lymphocytes, white blood cells, and few mesothelial and red blood cells. An exploratory laparotomy was performed; multiple grey-white miliary nodular lesions with a diameter ranging from 0.2 to 1.6 cm were observed on the surface of the left ovary, the uterus, the peritoneum, the omentum, and the small and large intestine. Histological examination of omental and peritoneal biopsies disclosed the presence of noncaseating, nonnecrotic, sarcoid type granulomas containing multinucleated giant cells (Figures 1 and 2). Moreover, the left ovary contained, upon sections, a whitish area consisting of cytopenic fibrous connective tissue (Figure 2) containing a single multinucleated giant cell (Figure 3), thus corresponding to a focus of fibrosed granuloma. Histochemical stains (PAS, Grocott's silver impregnation, and Ziehl-Neelsen) disclosed no fungi, argyrophilic, or acid-fast microorganisms.

The final histological diagnosis was granulomatous, sarcoid type inflammation. Evaluation by an expert pulmonologist revealed no evidence of lung sarcoidosis by CT scan and gallium scanning. The patient was further investigated in the direction of possible extra pulmonary involvement with no abnormal findings revealed. A decision to withhold further therapy was made. The patient is doing well after one year of follow-up without other complications.

## 3. Discussion

There was neither family history of sarcoidosis, nor history of immunodeficiency or biological factor therapy. Furthermore, the patient did not mention any occupational or environmental exposure in industrial work associated with beryllium; such exposure has been associated with granuloma formation, pulmonary sarcoidosis, and elevations of Serum Angiotensin-Converting Enzyme (SACE) activity [2]. No history of prior infections due to mycobacterial or cutibacteria (aka propionibacteria) was reported; such infections have been etiologically implicated in the pathogenesis of sarcoidosis [3, 4].

Histology confirmation is necessary for the diagnosis of sarcoidosis occurring outside usually affected organs, for example, the lung. In any case the physician needs to exclude the presence of infectious or neoplastic mimics of the disease. Sarcoidosis has been very rarely described to affect the female genital tract especially the ovaries and even more rarely in relation to a concomitant malignancy [5–7].

In our case peritoneal involvement was also noted which has been described more frequently [8–10]. In some reports sarcoidosis presented as a peritoneal malignancy [9, 11]. Involvement of both organs at the same time as was the case in our patient appears to be even rarer [12, 13]. In contrast to our case where weight loss and fatigue were the presenting symptoms, common features of female genital tract sarcoidosis include the presence of abdominal pain and abdominal distension as well as menstrual cycle disturbances. No radiological or nuclear findings are specific to this disease location [14]. The free fluid in the abdominal cavity directed the diagnostic work-up towards the presence of a neoplastic disease. In retrospect, the peritoneal involvement in our case explains the ascites; sarcoidosis of the peritoneum has been described to mimic other neoplasias such as peritoneal carcinomatosis [15].

With regard to the elevated biomarkers the abnormal CA-125 levels are explained by the presence of peritoneal involvement and ascites and have been previously described [10, 16]. It is well known that, besides gynaecological cancers, CA 125 levels are elevated also in various nongynecologic malignancies and other benign conditions [10]. In contrast, we identified only one other case where sarcoidosis of the breast was found after breast operation with a minimal elevation of CA 15.3 of 31.6 U/ml [17]. Abnormal CA 15.3 levels have been correlated with a wide range of cancers, including breast cancer; its use has been proposed as a disease marker in (fibrotic) pulmonary sarcoidosis that was not observed in our case [18].

In our report surgical intervention led to the correct diagnosis. Similarly, in previous reports, a provisional diagnosis of ovarian malignancy was excluded by exploratory laparotomy and histological confirmation of sarcoidosis [6, 13, 19].

In conclusion, we describe a rare case of ovarian, peritoneal, and omental sarcoidosis with no lung involvement and abnormally elevated CA 15-3 levels. Cancer biomarkers should not be used to exclude or confirm the diagnosis of cancer or sarcoidosis for the same matter. In our case their use misled the physicians in assuming the presence of a gynecological malignancy. Histological confirmation of the diagnosis is necessary in such difficult cases.
